# Diagnostic Performance of Quantitative and Qualitative Elastography for Axillary Lymph Node Metastasis in Breast Cancer: A Systematic Review and Meta-Analysis

**DOI:** 10.3389/fonc.2020.552177

**Published:** 2020-10-15

**Authors:** Xiao-wen Huang, Qing-xiu Huang, Hui Huang, Mei-qing Cheng, Wen-juan Tong, Meng-fei Xian, Jin-yu Liang, Wei Wang

**Affiliations:** ^1^Zhongshan Hospital of Traditional Chinese Medicine, Affiliated to Guangzhou University of Chinese Medicine, Zhongshan, China; ^2^Department of Medical Ultrasonics, Institute of Diagnostic and Interventional Ultrasound, The First Affiliated Hospital of Sun Yat-sen University, Guangzhou, China

**Keywords:** elastography, breast cancer, axillary lymph node metastasis, diagnose, meta-analysis

## Abstract

**Background:** Studies have shown inconsistent results regarding the diagnostic performance of ultrasound elastography for axillary lymph node metastasis (ALNM) in breast cancer. This meta-analysis aimed to estimate the diagnostic performance of ultrasound elastography (divided into quantitative and qualitative elastography) for ALNM in patients with breast cancer.

**Methods:** The PubMed and Embase databases were searched for eligible studies exploring the diagnostic performance of ultrasound elastography for ALNM in patients with breast cancer. The included studies were divided into quantitative and qualitative elastography groups to perform separate meta-analyses. The diagnostic performance was investigated with pooled sensitivity and specificity and diagnostic odds ratio (DOR) using a bivariate mixed-effects regression model. A summary receiver operating characteristic curve was constructed, and the area under the curve (AUC) was calculated.

**Results:** Seven and 11 studies were included in the quantitative and qualitative elastography meta-analyses, respectively. The pooled sensitivity and specificity, DOR, and AUC with their corresponding 95% confidence intervals were 0.82 (0.75, 0.87), 0.88 (0.78, 0.93), 33 (13, 83), and 0.89 (0.86, 0.91), respectively, for quantitative elastography and 0.81 (0.69, 0.89), 0.92 (0.79, 0.97), 46 (12, 181), and 0.92 (0.89, 0.94), respectively, for qualitative elastography. No significant publication bias existed. Fagan plots demonstrated good clinical utility. However, substantial heterogeneity existed among studies. Study design, measurement, and reference standard served as potential sources of heterogeneity for quantitative studies, which were measurement and reference standard for qualitative studies.

**Conclusions:** Both quantitative and qualitative elastography seem to be feasible, non-invasive diagnostic tools for ALNM in breast cancer. Nevertheless, the results must be interpreted carefully, paying attention to heterogeneity issues, especially for quantitative elastography studies.

## Introduction

The incidence of breast cancer is rising, and this disease is a serious threat to women's health. Pre-operative evaluation of axillary lymph node status can provide important reference values for determining the clinical stage of and treatment plans for breast cancer (1). Currently, axillary lymph node metastasis (ALNM) in breast cancer patients is diagnosed by axillary lymph node dissection, which may cause complications such as infection, nerve damage, or swelling of soft tissues in the axilla (2). Although sentinel lymph node biopsy is an important non-surgical alternative to axillary lymph node dissection, it is still an invasive procedure and inevitably produces false-negative results (3–5). Thus, it is necessary to explore non-invasive methods for the pre-operative assessment of ALNM.

Ultrasound elastography is a non-invasive detection method that can reflect information about the stiffness of the lesion (6, 7). Based on the fact that malignant lesions are usually harder than normal tissue, many studies have explored the diagnostic value of ultrasound elastography for ALNM in breast cancer (8–12). Some studies have demonstrated that ultrasound elastography is helpful in the pre-operative evaluation of axillary lymph node status (8–10). However, some researchers have reported that the diagnostic performance of ultrasound elastography in ALNM is insufficient (11, 12). Because of these conflicting results, it is necessary to perform a meta-analysis to assess the diagnostic value of ultrasound elastography for ALNM in breast cancer.

According to different imaging principles, ultrasound elastography can be methodologically divided into quantitative and qualitative elastography to assess tissue stiffness (6, 7). Quantitative elastography, mainly shear wave imaging, uses short-duration acoustic radiation forces to generate small localized tissue displacements (1–10 μm), which cause shear wave propagation and are tracked to calculate shear wave velocity or converted to Young's modulus to reflect tissue stiffness. Qualitative elastography, mainly strain imaging, reflects tissue stiffness through the color gradation superimposed on grayscale ultrasound images. Thus, to explore the impact of different imaging principles, we simultaneously investigated the diagnostic performance of quantitative and qualitative elastography for ALNM in breast cancer in this meta-analysis.

## Materials and Methods

### Search Strategy

We searched the PubMed and Embase databases for studies that assessed the diagnostic performance of ultrasound elastography for ALNM in breast cancer through December 2019. The main search terms were elastography/stiffness, breast cancer, and lymph node/metastasis. The detailed search strategy is shown in the Supplementary Material. The search procedure was performed and confirmed by two investigators (X-wH and Q-xH).

### Inclusion and Exclusion Criteria

Studies that met the following criteria were included in the meta-analysis: (1) the study was published in English; (2) ultrasound elastography was used to assess axillary lymph node status; (3) the study population consisted of at least 30 confirmed breast cancer patients; and (4) true-positive (TP), false-positive (FP), false-negative (FN), and true-negative (TN) data could be directly or indirectly obtained to construct a diagnostic 2 × 2 table. Duplicate studies, reviews, letters, editorials, case reports, non-human studies, and unrelated studies were excluded. The reference lists of the included studies were reviewed to identify other potentially eligible studies. Two investigators (HH and MC) independently reviewed and selected the studies. In case of disagreement, a consensus was required to reach a decision.

### Data Extraction and Quality Assessment

The following data were extracted: first author, published year, country, study design (prospective or retrospective), number of lymph nodes, number of patients, mean age, elastography method, region of interest (ROI) position and size, measurement index, cutoff value, area under the receiver operating characteristic curve (AUC), sensitivity, specificity, TP, FP, FN, TN, and reference standard. If more than one measurement index from the same elastography was reported, only the measurement index with the highest diagnostic performance was extracted. Study quality was assessed using the Quality Assessment of Studies of Diagnostic Accuracy included in Systematic Review (QUADAS-2 tool) (13). This tool was used to assess the quality of diagnostic tests with respect to the following four aspects: patient selection, index test, reference standard, and flow and timing. Each aspect was evaluated based on the risk of bias (low, high, or unclear), with the first three aspects related to applicability. Two investigators (W-jT and M-fX) were assigned to data extraction and quality assessment. When disagreement arose, a consensus was required to reach a decision.

### Statistical Analysis

All statistical analyses were performed using the MIDAS module of Stata, Version 12.0 (Stata, College Station, TX, USA), except for the quality assessment graphs, which were plotted using Review Manager, Version 5.3 (Copenhagen: The Nordic Cochrane Center, The Cochrane Collaboration, 2014). All analyses were applied to the quantitative and qualitative elastography meta-analyses.

#### Pooled Diagnostic Performance

A bivariate mixed-effects regression model was used to calculate the pooled sensitivity, specificity, positive likelihood ratio (PLR), and negative likelihood ratio (NLR). The diagnostic odds ratio (DOR), the ratio of positivity in diseased patients to positivity in non-diseased patients, was calculated to indicate the diagnostic performance (14). A summary receiver operating characteristic (SROC) curve was plotted, and the AUC with a 95% confidence interval (CI) was calculated to quantitatively assess the pooled result.

#### Publication Bias

It is necessary to assess publication bias for the included studies because studies that report positive results may be easier to publish. Publication bias was assessed by testing the asymmetry of Deeks' funnel plot [the inverse of the square root of the effective sample size (1/rootESS) vs. the natural logarithm of the DOR (lnDOR)]. *P* < 0.05 for the slope coefficient indicates significant asymmetry, which thus indicates significant publication bias (15).

#### Heterogeneity Assessment

Heterogeneity was assessed using the inconsistency index *I*^2^; a value >50% was considered substantial heterogeneity. If heterogeneity existed, subgroup analysis and meta-regression analysis were performed to explore the potential sources of heterogeneity with the following factors: country, study design (prospective or retrospective), measurement index, reference standard, *ex/in vivo* research, ROI size, and publication form (conference abstract or full text).

#### Clinical Utility

The clinical utility was assessed using a Fagan plot (16), which provided the post-test probability (*P*_post_) of ALNM when pre-test probabilities (*P*_pre_, suspicion of ALNM) were provided. *P*_post_ was calculated from the likelihood ratio (LR) using Bayes's theorem, with *P*_post_ = (LR × *P*_pre_)/[(1 − *P*_pre_) × (1 − LR)]. In this meta-analysis, a *P*_pre_ of 50% was provided to determine the corresponding *P*_post_ of ALNM.

## Results

### Search Results

**Figure 1:**
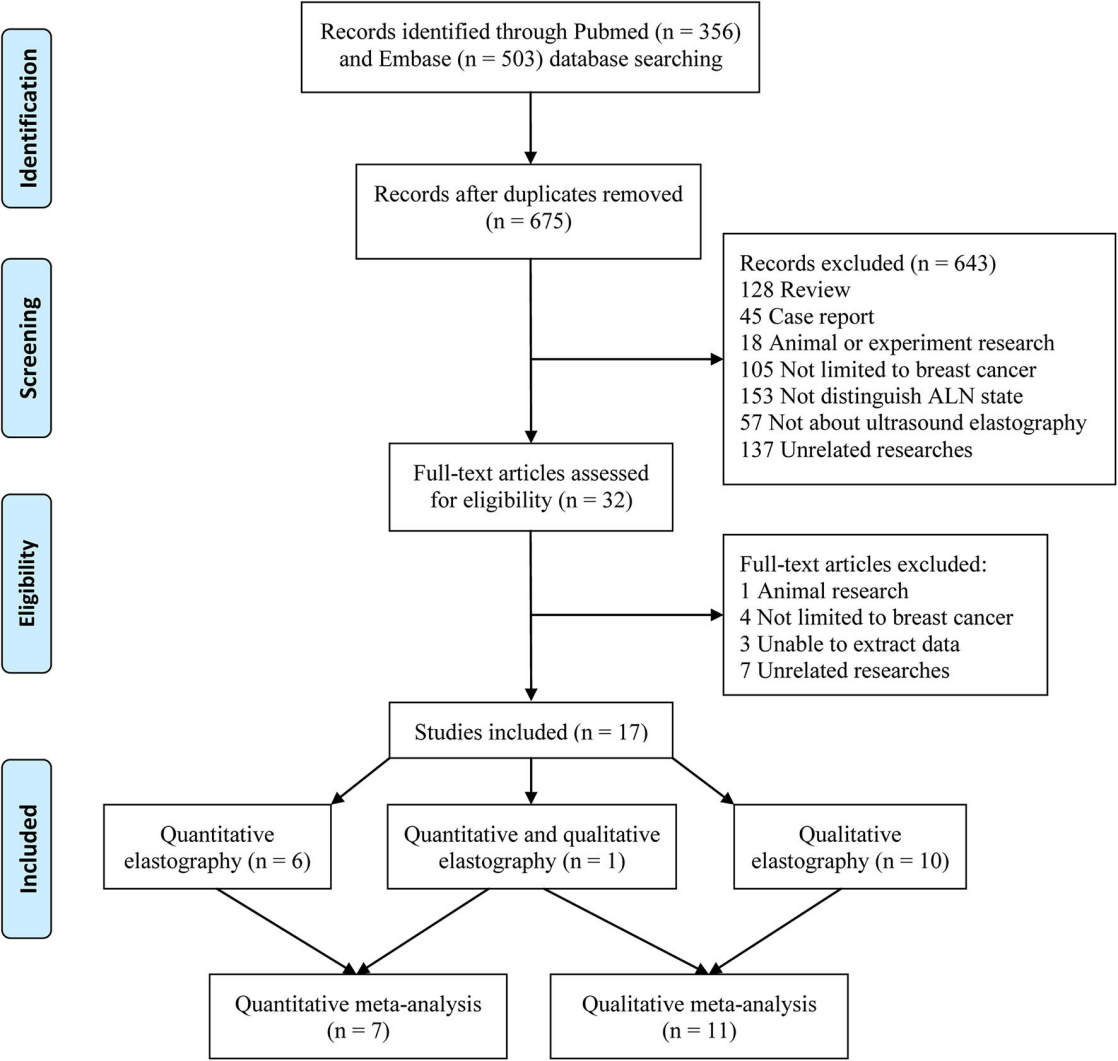
Flowchart of eligible studies.

A total of 356 and 503 studies were initially searched from the PubMed and Embase databases, respectively. After removing duplicates, 675 studies were further reviewed. After reviewing the titles and abstracts, 643 studies not related to the topic were removed. Then, the full texts of 32 studies were further reviewed, and 15 studies were removed (one animal study, four studies not limited to breast cancer patients, three studies from which the data could not be extracted, and seven unrelated studies). Finally, 17 studies were included in this meta-analysis. Among the 17 included studies, six reported only quantitative elastography measurements (17–22), ten reported only qualitative elastography measurements (9–12, 23–28), and one reported both quantitative and qualitative elastography measurements (8). Therefore, the seven studies that reported quantitative elastography measurements were included in the quantitative elastography meta-analysis (8, 17–22). The 11 studies that reported qualitative elastography measurements were included in the qualitative elastography meta-analysis (8–12, 23–28). The inclusion and exclusion processes are summarized in Figure 1.

### Study Characteristics and Quality Assessment

The main characteristics of the included studies are displayed in Table 1. There were 827 (251 metastatic, 576 benign) and 1,072 (576 metastatic, 496 benign) lymph nodes included in the quantitative and qualitative elastography meta-analyses, respectively. In the quantitative studies, two were *ex vivo* assessments of lymph nodes (18, 20). In the qualitative studies, two were published as conference abstracts (9, 11). One study compared a new qualitative pattern classification for shear wave elastography (SWE) to quantitative SWE parameters (8). Therefore, this study was included in the quantitative elastography meta-analysis (including the quantitative SWE parameters) and the qualitative elastography meta-analysis (including the qualitative SWE pattern classification). The QUADAS-2 tool showed that the included studies generally had good methodological quality (Table 2), which was intuitively displayed in the corresponding quality assessment graphs (Supplementary Material, Supplementary Figures 1, 2).

**Table 1:** Characteristics of the included studies.

	**Author, year**	**Participant country**	**Study design**	**Lymph nodes, *n***	**Patients, *n***	**Mean age**	**Elastography method**	**ROI position**			**ROI size**
Quantitative studies	Luo, 2019a (8)	China	Prospective	121	118	46.68	SWE	First ROI: stiffest region of target ALN Second ROI: surrounding fatty tissue			1-mm-diameter circle
	Seo, 2018 (17)	Korea	Retrospective	54	53	NA	SWE	First ROI: stiffest region of target ALN Second ROI: surrounding fatty tissue			3-mm-diameter circle
	Bae, 2018^c^ (18)	Korea	Prospective	228	55	49	SWE	First ROI: stiffest region of target ALN Second ROI: surrounding fatty tissue			2-mm-diameter circle
	Youk, 2017 (19)	Korea	Retrospective	130	130	49.4	SWE	First ROI: stiffest region of target ALN Second ROI: surrounding fatty tissue			2-mm-diameter circle
	Kilic, 2016^c^ (20)	Turkey	Prospective	64	30	NA	SWE	Stiffest region for the hilar and cortical regions of target ALN			2-mm-diameter circle
	Tamaki, 2013 (21)	Japan	NA	149	149	57	VTQ	Fastest velocities of the central and cortical areas of ALN			5 × 5 mm
	Tourasse, 2012 (22)	France	Prospective	81	NA	NA	SWE	First ROI: stiffest region of target ALN Second ROI: surrounding fatty tissue			2-mm-diameter circle
Qualitative studies	Luo, 2019b (8)	China	Prospective	121	118	46.68	SWE	Comprise target ALN and surrounding tissue			NA
	Zhao, 2018 (23)	China	Prospective	78	78	52.47	SE	Shallow layers with subcutaneous fat and part of deep layers excluding axillary vessel and pectoralis muscles; comprise target ALN and surrounding tissue			Two or more times target ALN
	Xu, 2018 (24)	China	Prospective	97	92	51	SE	Comprise target ALN and surrounding tissue			Greater than or equal to two times larger than target ALN
	Chang, 2018 (25)	China	Retrospective	140	140	55.3	SE	Comprise target ALN and surrounding tissue			NA
	Zhang, 2017 (26)	China	Retrospective	161	158	55.2	SE	Comprise target ALN and surrounding tissue			NA
	Leong, 2017^d^ (9)	USA	Retrospective	70	70	NA	NA	NA			NA
	Kleditzsch, 2017^d^ (11)	Germany	Prospective	97	97	NA	SE	NA			NA
	Park, 2014 (12)	USA	Prospective	104	101	55	SE	Comprise target ALN and surrounding tissue			3.0-cm-wide and 2.5-cm-deep rectangular box
	Tsai, 2013 (10)	China	Prospective	90	89	51	SE	Comprise target ALN and surrounding subcutaneous fat and muscle in the same proportion, but excluded other tissues			NA
	Taylor, 2011 (27)	United Kingdom	Prospective	50	50	57	SE	Comprise target ALN and surrounding tissue			NA
	Choi, 2011 (28)	Korea	Retrospective	64	62	53	SE	Comprise target ALN and surrounding tissue, exclude pectoralis muscles and axillary vessels.			NA
	**Author, year**	**Measurement index**	**Cutoff value**	**AUC**	**Sensitivity (%)**	**Specificity (%)**	**TP**	**FP**	**FN**	**TN**	**Reference standard**
Quantitative studies	Luo, 2019a (8)	Emean	>26.9 kPa	0.946	86.7	96.7	52	2	8	59	Biopsy + surgery
	Seo, 2018 (17)	Emax	≥20.9 kPa	0.887	82.35	95.00	28	1	6	19	Biopsy + surgery
	Bae, 2018^c^ (18)	Eratio	>2.37	0.831	70.7	88.8	29	21	12	166	Surgery
	Youk, 2017 (19)	Eratio	>2.7	0.950	90.8	93.9	59	4	6	61	Biopsy + surgery
	Kilic, 2016^c^ (20)	Cortical Emean	>14.75 kPa	0.786	75	83	9	9	3	43	Surgery
	Tamaki, 2013 (21)	Shear wave speed	>1.44 m/s	NA	82.8	69.6	23	37	5	84	OSNA
	Tourasse, 2012 (22)	Emean	NA	0.762	81.98	74.22	9	18	2	52	Surgery
Qualitative studies	Luo, 2019b (8)	Color pattern^e^	Color pattern 2	0.983	96.7	100	58	0	2	61	Biopsy + surgery
	Zhao, 2018 (23)	Elasticity score	≥score 3	0.898	86.4	85.3	38	5	6	29	Surgery
	Xu, 2018 (24)	Elasticity score	≥score 3	0.916	78	93	41	3	11	42	Biopsy
	Chang, 2018 (25)	Hard area ratio	≥50%	NA	60.26	96.77	47	2	31	60	Biopsy + surgery
	Zhang, 2017 (26)	Hard area ratio	>50%	0.683	38.0	98.6	35	1	57	68	Biopsy + surgery
	Leong, 2017^d^ (9)	Elastographic size ratio	NA	NA	93.5	100	43	0	3	24	Biopsy + surgery
	Kleditzsch, 2017^d^ (11)	SR max	NA	0.68	74.1	50.9	24	31	9	33	Surgery
	Park, 2014 (12)	Hard area ratio	≥50%	0.616	68.5	54.5	47	16	22	19	Biopsy + surgery
	Tsai, 2013 (10)	Hard area ratio	>50%	0.907	86	90	43	4	7	36	FNA cytology
	Taylor, 2011 (27)	Elasticity score	≥score 3	0.90	90	86	19	4	2	25	Biopsy + surgery
	Choi, 2011 (28)	Elasticity score	≥score 3	0.784	80.7	66.7	25	11	6	22	Biopsy + surgery

a, b, *Same study reported both quantitative and qualitative elastography methods*.

c *Lymph nodes in the two researches were ex vivo*.

d *The two researches published as conference abstract*.

e *A new qualitative pattern classification of SWE proposed by the author*.

*NA, data not available; SWE, shear wave elastography; VTQ, virtual touch quantification; SE, strain elastography; Emean, mean elasticity; Emax, maximum elasticity; Eratio, elasticity ratio (lesion-to-fat); SR, strain ratio; AUC, area under curve; TP, true positive; FP, false positive; FN, false negative; TN, true negative; OSNA, one-step nucleic acid amplification; FNA, fine needle aspiration; ROI, region of interest; ALN, axillary lymph node*.

**Table 2:** Quality assessment of the included studies.

	**Author, year**	**Risk of bias**				**Applicability concerns**		
		**Patient selection**	**Index test**	**Reference standard**	**Flow and timing**	**Patient selection**	**Index test**	**Reference standard**
Quantitative studies	Luo, 2019a (8)	Unclear	Low	Low	Unclear	Low	Low	Low
	Seo, 2018 (17)	High	Low	Low	Unclear	Unclear	Low	Low
	Bae, 2018 (18)	Low	Low	Low	Unclear	Low	Low	Low
	Youk, 2017 (19)	Low	Low	Unclear	Unclear	Low	Low	Unclear
	Kilic, 2016 (20)	Unclear	Low	Low	Low	Low	Low	Low
	Tamaki, 2013 (21)	Unclear	Low	Unclear	Low	Low	Low	High
	Tourasse, 2012 (22)	High	Low	Low	Unclear	High	Low	Low
Qualitative studies	Luo, 2019b (8)	Unclear	Low	Low	Unclear	Low	Low	Low
	Zhao, 2018 (23)	Low	Low	Low	Low	Low	Low	Low
	Xu, 2018 (24)	Unclear	Low	Low	Unclear	Unclear	Low	Low
	Chang, 2018 (25)	Low	Low	Low	Unclear	Low	Low	Low
	Zhang, 2017 (26)	Low	Low	Low	Unclear	Low	Low	Low
	Leong, 2017 (9)	Unclear	Unclear	Low	Unclear	Unclear	Unclear	Low
	Kleditzsch, 2017 (11)	Unclear	Low	Low	Low	Unclear	Low	Low
	Park, 2014 (12)	Low	Low	Low	Low	Low	Low	Low
	Tsai, 2013 (10)	Unclear	Unclear	Unclear	Low	Unclear	Low	High
	Taylor, 2011 (27)	Unclear	Low	Low	Unclear	Unclear	Low	Low
	Choi, 2011 (28)	Low	Low	Unclear	Low	Low	Low	Low

*a, b, Same study reported both quantitative and qualitative elastography methods*.

### Diagnostic Performance

**Figure 2:**
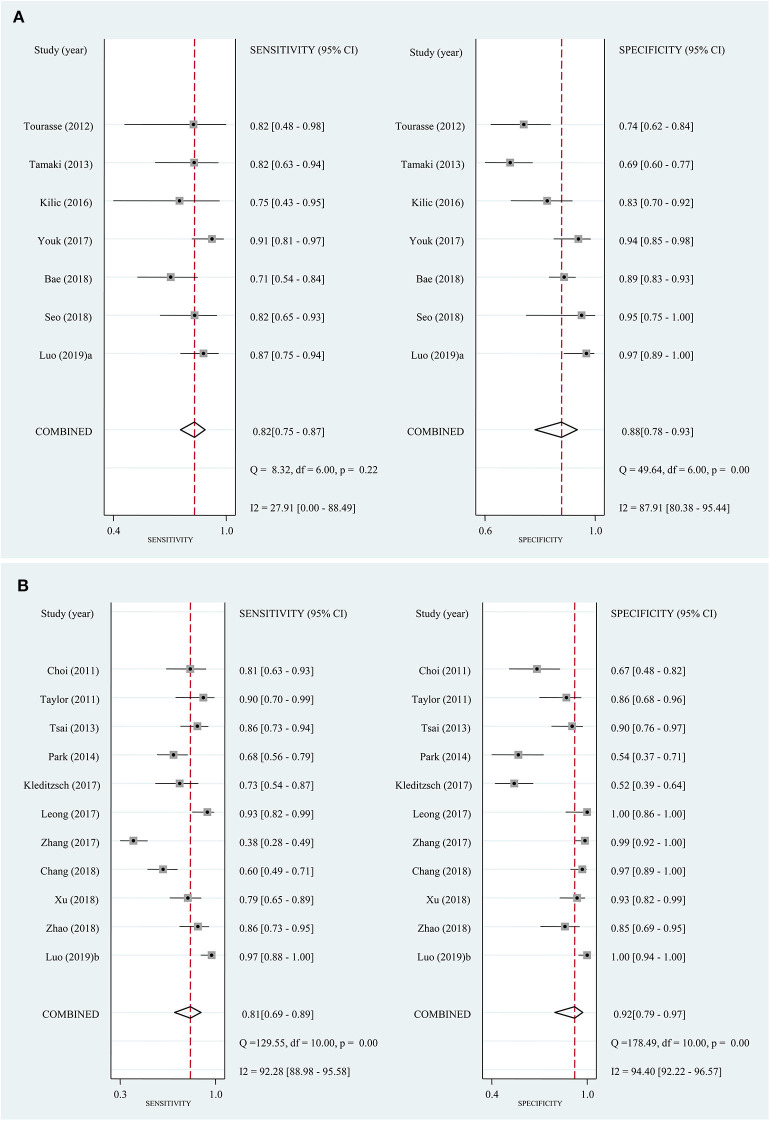
Forest plots of sensitivity and specificity for the included studies. Bivariate mixed-effects regression model was used to calculate the pooled sensitivity and specificity. **(A)** Quantitative elastography studies; **(B)** qualitative elastography studies. CI, confidence interval.

**Figure 3:**
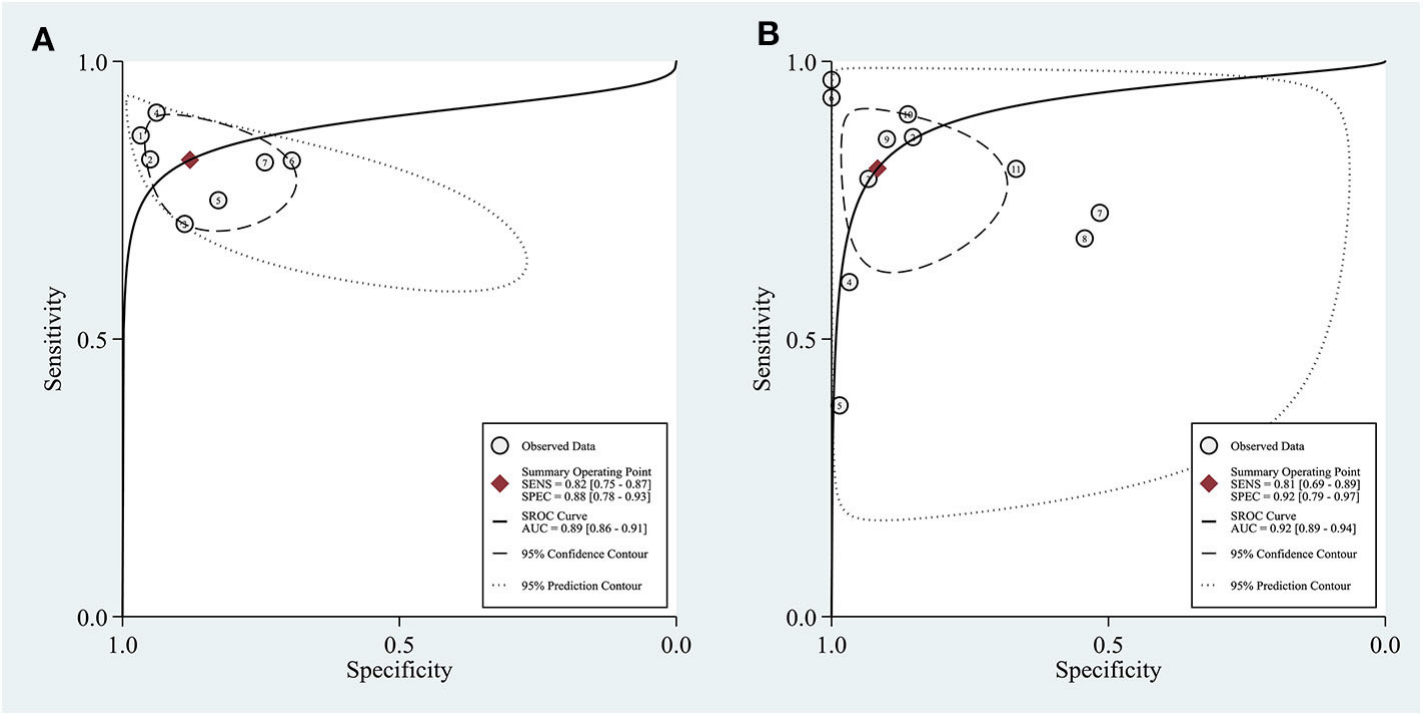
Sensitivity and specificity plotted in summary receiver operating characteristic (SROC) curves of the included studies. Area under the curve (AUC) was calculated to quantitatively assess the pooled result. **(A)** Quantitative elastography studies; **(B)** qualitative elastography studies. SENS, sensitivity; SPEC, specificity.

In the seven studies included in the quantitative elastography meta-analysis, the sensitivity and specificity for ALNM diagnosis ranged from 70.7 to 90.8% and 69.6 to 96.7%, respectively (Table 1). In the 11 studies included in the qualitative elastography meta-analysis, the sensitivity and specificity for ALNM diagnosis ranged from 38 to 96.7% and 50.9 to 100%, respectively (Table 1). The sensitivity and specificity of the pooled results are displayed in the forest plots (Figure 2). For the quantitative elastography studies, the pooled sensitivity, specificity, PLR, NLR, and DOR with their corresponding 95% CIs were 0.82 (0.75, 0.87), 0.88 (0.78, 0.93), 6.8 (3.6, 12.7), 0.20 (0.14, 0.29), and 33 (13, 83), respectively; the corresponding values for the quantitative studies were 0.81 (0.69, 0.89), 0.92 (0.79, 0.97), 9.7 (3.4, 27.4), 0.21 (0.12, 0.36), and 46 (12, 181). The areas under the SROC curves for the quantitative and qualitative elastography studies were 0.89 (0.86, 0.91) and 0.92 (0.89, 0.94), respectively (Figure 3).

### Publication Bias

**Figure 4:**
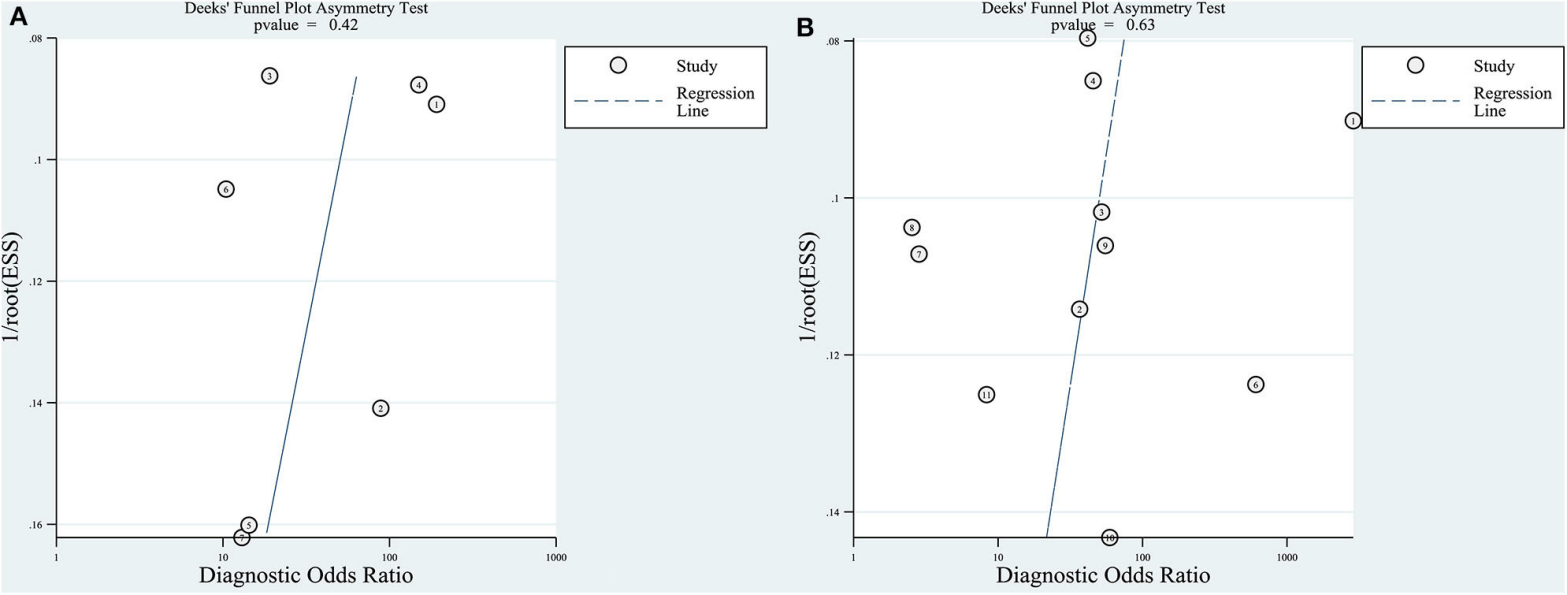
Deeks' funnel plot asymmetry test for testing publication bias. Publication bias was assessed by testing the asymmetry of Deeks' funnel plot [the inverse of the square root of the effective sample size (1/rootESS) vs. the natural logarithm of the DOR (lnDOR)]. *P* < 0.05 for the slope coefficient indicates significant asymmetry, i.e., significant publication bias. **(A)** Quantitative elastography studies; **(B)** qualitative elastography studies.

For the quantitative and qualitative elastography meta-analysis, *P*-values for testing the asymmetry of the Deeks' funnel plots were 0.42 and 0.63, respectively, which meant that there was no significant publication bias among the included studies (Figure 4).

### Heterogeneity Assessment

For the quantitative and qualitative elastography meta-analysis, the values of *I*^2^ and the corresponding 95% CIs were 66% (24%, 100%) and 97% (95%, 99%), respectively, which indicated substantial heterogeneity among the included studies. The results of subgroup analysis and meta-regression analysis for country, study design, measurement index, reference standard, *ex/in vivo* research, ROI size, and publication form (conference abstract or full text) are shown in Table 3. According to meta-regression analysis, study design (*P* < 0.01), measurement (*P* < 0.01), and reference standard (*P* < 0.01) were potential sources of heterogeneity for quantitative studies, which were measurement (*P* < 0.01) and reference standard (*P* < 0.01) for qualitative studies.

**Table 3:** Subgroup analysis and meta-regression analysis.

	Subgroup*	**Studies, *n***	**Sensitivity (95% CI)**	***P-sen***	**Specificity (95% CI)**	***P-spe***	***P-meta***
Quantitative studies	Total	7	0.82 (0.75–0.87)		0.88 (0.78–0.93)		
	**Country**			0.02		<0.01	0.22
	Korea	3	0.83 (0.75–0.91)		0.93 (0.87–0.99)		
	Others	4	0.82 (0.74–0.91)		0.82 (0.72–0.92)		
	**Study design**			<0.01		0.05	<0.01
	Prospective	4	0.78 (0.69–0.87)		0.87 (0.80–0.95)		
	Retrospective	2	0.88 (0.81–0.95)		0.94 (0.87–1.00)		
	**Measurement**			0.20		0.03	<0.01
	Emean	3	0.81 (0.68–0.93)		0.86 (0.76–0.95)		
	Eratio	2	0.83 (0.71–0.94)		0.92 (0.86–0.99)		
	**Reference standard**			<0.01		<0.01	<0.01
	All surgery	3	0.75 (0.63–0.86)		0.83 (0.77–0.90)		
	Partial surgery	3	0.87 (0.82–0.93)		0.95 (0.92–0.99)		
	***Ex/in vivo*** **research**			<0.01		0.37	0.09
	*Ex vivo*	2	0.71 (0.58–0.84)		0.87 (0.73–1.00)		
	*In vivo*	5	0.86 (0.80–0.91)		0.88 (0.79–0.97)		
	ROI size			0.01		0.31	0.85
	2 mm circle	4	0.81 (0.72–0.89)		0.87 (0.77–0.97)		
	others	3	0.84 (0.76–0.92)		0.89 (0.78–1.00)		
Qualitative studies	Total	11	0.81 (0.69–0.89)		0.92 (0.79–0.97)		
	**Country**			0.23		0.10	0.05
	China	6	0.79 (0.64–0.93)		0.96 (0.92–1.00)		
	Others	5	0.84 (0.70–0.97)		0.76 (0.58–0.95)		
	**Study design**			0.77		0.37	0.10
	Prospective	7	0.85 (0.76–0.94)		0.88 (0.74–1.00)		
	Retrospective	4	0.71 (0.51–0.90)		0.97 (0.90–1.00)		
	**Measurement**			0.62		0.36	<0.01
	Elasticity score	4	0.84 (0.75–0.94)		0.85 (0.70–1.00)		
	Hard area ratio	4	0.64 (0.50–0.77)		0.92 (0.82–1.00)		
	**Reference standard**			0.96		0.31	<0.01
	All surgery	2	0.81 (0.56–1.00)		0.72 (0.21–1.00)		
	Partial surgery	7	0.80 (0.66–0.94)		0.95 (0.87–1.00)		
	**Abstract/full text**			0.82		0.72	0.77
	Abstract	2	0.86 (0.69–1.00)		0.89 (0.62–1.00)		
	Full text	9	0.79 (0.68–0.90)		0.92 (0.84–1.00)		

* *Some studies were not classified into subgroups because the number was <2*.

*P-sen, P-value for sensitivity; P-spe, P-value for specificity; P-meta, P-value after meta-regression analysis*.

### Clinical Utility

**Figure 5:**
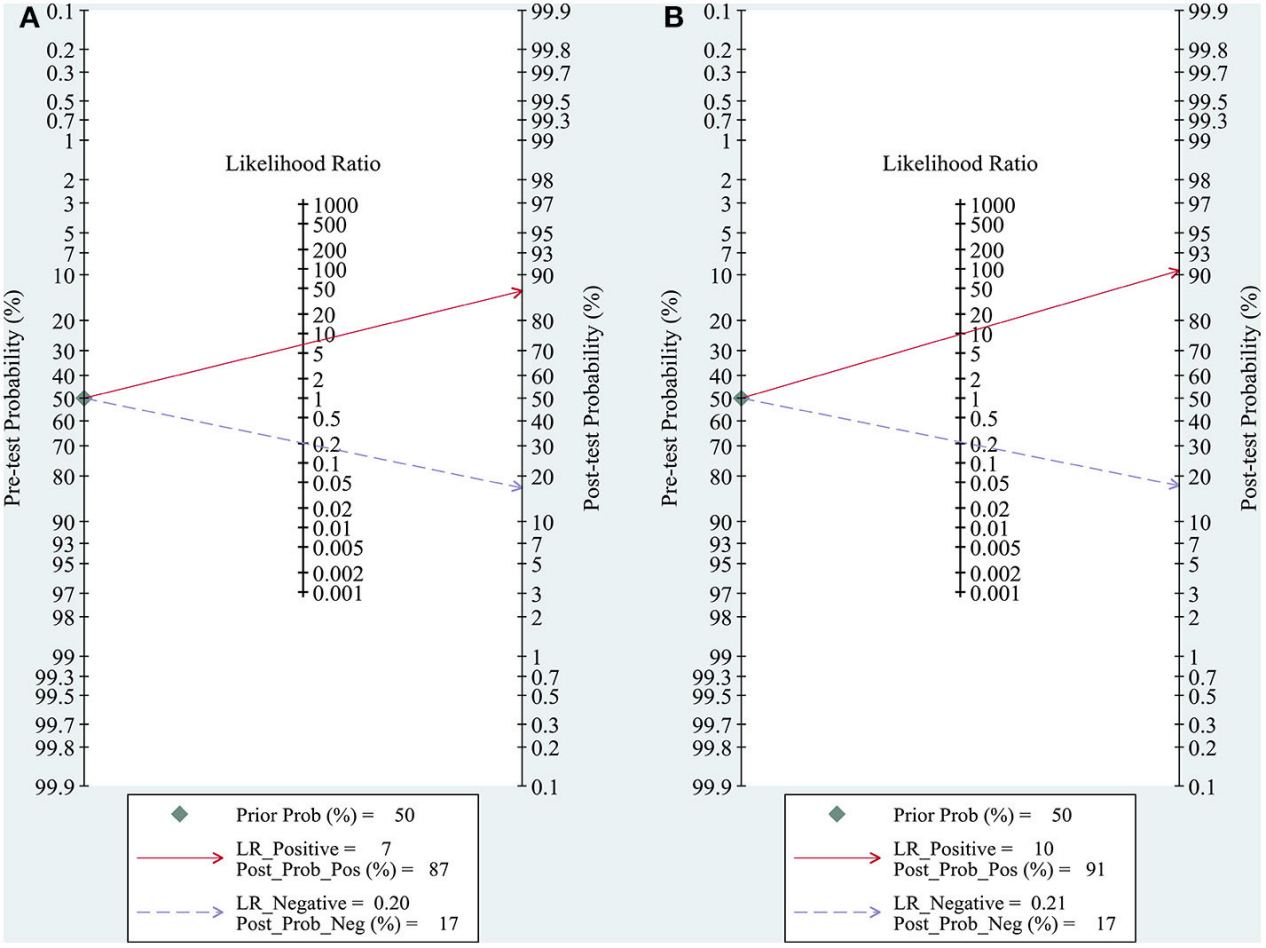
Fagan plots for assessing the clinical utility of quantitative **(A)** and qualitative **(B)** elastography to diagnose axillary lymph node metastasis (ALNM) in breast cancer. Fagan plot provided the post-test probability (*P*_post_) of ALNM when pre-test probabilities (*P*_pre_, suspicion of ALNM) were provided. *P*_post_ was calculated from the likelihood ratio (LR) using Bayes's theorem, with *P*_post_ = (LR × *P*_pre_)/[(1 – *P*_pre_) × (1 – LR)]. In this meta-analysis, a *P*_pre_ of 50% was provided to determine the corresponding *P*_post_ of ALNM.

Both quantitative and qualitative elastography were demonstrated to have good clinical utility in the diagnosis of ALNM in breast cancer (Figure 5). At a *P*_pre_ of 50%, the positive and negative *P*_post_ values were 87 and 17% for quantitative elastography (Figure 5A) and 91 and 17% for qualitative elastography, respectively (Figure 5B).

## Discussion

We performed this meta-analysis to investigate the diagnostic performance of quantitative and qualitative elastography for ALNM in breast cancer. Elastography was found to have good diagnostic performance in diagnosing ALNM, with a pooled sensitivity and specificity of 0.82 and 0.88 for quantitative elastography and of 0.81 and 0.92 for qualitative elastography, respectively. Both quantitative and qualitative elastography were demonstrated to have good clinical utility in the diagnosis of ALNM in breast cancer.

The present meta-analysis encompassed both quantitative and qualitative elastography, which assess tissue stiffness based on different imaging principles (6, 7). Quantitative and qualitative elastography have comparable performance in diagnosing ALNM because the pooled sensitivity, specificity, and AUC of the two techniques were similar. In addition, the Fagan plots demonstrated the good clinical utility of quantitative and qualitative elastography in diagnosing ALNM in breast cancer (Figure 5). Thus, the differences between the imaging principles of the quantitative and qualitative elastography seemed to have not led to significant differences in diagnostic performance. However, no studies have directly compared the diagnostic performance of the two methods for ALNM except for one current study, which claimed that qualitative SWE classification showed better diagnostic performance than quantitative SWE parameters (8). However, the proposed qualitative SWE classification method was a new qualitative pattern classification first proposed by the authors, which differed from common qualitative elastography (strain imaging). On the other hand, compared with quantitative elastography, the qualitative one is simpler with relatively unified principle and measurement index based on strain pattern (7). In addition, regarding performance as the sensitivity and specificity as shown, in the forest plot (Figure 2), variations at each study of quantitative elastography is significantly larger than the qualitative one. Therefore, future studies are required to compare the differences in the diagnostic performance between quantitative and qualitative elastography for ALNM in breast cancer.

Quantitative elastography assesses tissue stiffness using shear wave velocity or Young's modulus. Among the seven included quantitative elastography studies, six investigated the diagnostic performance of SWE in ALNM. Various quantitative elasticity measurements can be obtained with SWE, including maximum elasticity (Emax), mean elasticity (Emean), minimum elasticity (Emin), the standard deviation of the ROI, and the ratio of elasticity (Eratio) of the lesion to the surrounding normal tissue. To maximize the diagnostic performance, if a study reported more than one quantitative elasticity measurement, only the measurement with the highest diagnostic performance was extracted to pool the estimates. Finally, heterogeneity was found among the studies, with an *I*^2^ of 66% (95% CI, 24, 100%). Subgroup analysis indicated a significant difference between the pooled specificity of Emean and Eratio. Meta-regression analysis suggests that the elasticity measurements were one possible source of heterogeneity. In addition, other factors, including the study design and reference standard, also served as potential sources of heterogeneity among the included quantitative elastography studies (Table 3). These results suggest that the heterogeneity among the quantitative elastography studies may be the result of many factors. Therefore, prospective multicenter studies controlling confounding variables as much as possible are necessary to explore the diagnostic performance of quantitative elastography in ALNM.

Qualitative elastography assesses tissue stiffness using the hard area ratio of the lymph nodes. The common two practices are to directly evaluate the hard area ratio or to convert the hard area ratio to an elasticity score to assess the stiffness of lymph nodes. In the qualitative elastography meta-analysis, four studies directly evaluated the hard area ratio (10, 12, 25, 26), while four others converted the hard area ratio as the elasticity score (23, 24, 27, 28). We found that the optimal cutoff values of the hard area ratio were all 50%, and the optimal cutoff values of elasticity score were all 3. A score of 3 corresponded to a hard area ratio ≥50% in three studies (23, 24, 27) and to a hard area ratio ≥45% in one study (28). Therefore, the optimal cutoff values of the hard area ratio and elasticity score were similar, which did not result in significant heterogeneity. Subgroup analysis also indicated no significant difference in the sensitivity and specificity between studies using the hard area ratio or the elasticity score as qualitative measurements (Table 3). However, meta-regression analysis indicated that measurement was a potential (*P* < 0.01) source of heterogeneity (Table 3). We thought that the subgroup analysis result of measurement may have been affected by other confounding variables. In fact, the heterogeneity among the studies may have been caused by many factors, such as the search strategy, inclusion, and exclusion criteria, parameter measurement, statistical analysis, and characteristics of each study (29). There may be many explanations for the sources of heterogeneity. We hypothesized that interobserver differences in the naked-eye assessment of the hard area ratio, which may be affected by the experience of the operators, the examination process, the machine parameters, etc., may be one possible reason. Further studies to explore the impact of individual differences in the diagnostic performance of qualitative elastography may be helpful to explain the observed heterogeneity.

Our study had several limitations. First, studies published in languages other than English were not included in this meta-analysis. However, this did not seem to produce significant publication bias in either the quantitative or the qualitative elastography studies (Figure 4). Second, substantial heterogeneity was found among both the quantitative and qualitative elastography studies, as we clarified in the above discussion, which would limit recommending their integration into clinical practice. The above two issues must be interpreted carefully, paying attention to the small number of quantitative studies. Theoretically, the funnel plot was invalid because the accuracy is low when fewer than 10 studies are available for the quantitative summary (30). Thus, the number of quantitative studies (seven) is too small to carry out statistical evaluation such as publication bias and analysis for the heterogeneity. Besides, according to the results of quality assessment (Table 2 and Supplementary Figure 1), two studies with high risk of bias are low in quality (17, 22), which possibly reduced the accuracy of bias assessment. Third, the ultrasound machines of the included studies were not exactly the same. Technological improvements or system errors may lead to measurement bias. In addition, the assessment and diagnostic performance of ultrasound elastography rely on the experience and skills of the operators as well as the operating procedures. All elastography imaging techniques have a learning curve for implementation to daily practice. Therefore, particularly for beginner operators, the diagnostic performance of these imaging tools may not be satisfactory as concluded in this meta-analysis. All these limitations of ultrasound elastography serve as potential sources of heterogeneity.

In conclusion, the present meta-analysis suggests high diagnostic performance of ultrasound elastography for ALNM in breast cancer. Both quantitative and qualitative elastography were carried out with high and comparable sensitivity and specificity. However, because of the substantial heterogeneity among these studies, evidence of data reliability is still insufficient. The results must be interpreted carefully, paying particular attention to heterogeneity issues, especially for quantitative elastography studies. Adequate method for higher accuracy of ALNM is needed in the future. Prospective multicenter population-based trials are necessary to confirm the diagnostic value of ultrasound elastography for ALNM in breast cancer.

## Data Availability Statement

All datasets generated for this study are included in the article/Supplementary Material.

## Author Contributions

JL and WW conceived and designed the study strategy. XH and QH worked for study search. HH and MC worked for study selection. WT and MX extracted data from each included study and assessed the study quality. XH prepared the tables and all figures. WW worked as the supervisor and made arbitration for all possible disagreements. All authors have read and approved the content.

## Conflict of Interest

The authors declare that the research was conducted in the absence of any commercial or financial relationships that could be construed as a potential conflict of interest.
