# Supplementary material for: Diagnostic Performance of Quantitative and Qualitative Elastography for Axillary Lymph Node Metastasis in Breast Cancer: A Systematic Review and Meta-Analysis
Source: Front Oncol. 2020 Oct 15;10:552177. doi: 10.3389/fonc.2020.552177 (PMC7593678; doi:10.3389/fonc.2020.552177)

Search strategy

Pubmed

① breast cancer	("Breast Neoplasms"[Mesh]) OR ((((breast[Title/Abstract]) OR mammary[Title/Abstract])) AND (("Neoplasms"[Mesh]) OR (((((Neoplasm*[Title/Abstract]) OR Tumor*[Title/Abstract]) OR Cancer*[Title/Abstract]) OR Carcinoma*[Title/Abstract]) OR Malignan*[Title/Abstract])))
② elastography/stiffness	(((((((((stiff*[Title/Abstract]) OR elastic*[Title/Abstract]) OR elastogra*[Title/Abstract]) OR fibroscan[Title/Abstract]) OR acoustography[Title/Abstract]) OR acoustographies[Title/Abstract]) OR sonoelastography[Title/Abstract]) OR sonoelastographies[Title/Abstract]) OR acoustic Radiation Force Impulse[Title/Abstract]) OR ARFI[Title/Abstract])
③ lymph node/metastasis	(((("Neoplasm Metastasis"[Mesh]) OR "Lymphatic Metastasis"[Mesh]) OR "Lymph Nodes"[Mesh])) OR ((((lymph[Title/Abstract]) OR lymphatic[Title/Abstract]) OR metastases[Title/Abstract]) OR metastasis[Title/Abstract])
Final search strategy	① AND ② AND ③

Final record: 356 studies.

Embase

breast cancer	#1	breast:ab,ti OR mammary:ab,ti
	#2	neoplasm*:ab,ti OR tumor*:ab,ti OR cancer*:ab,ti OR carcinoma*:ab,ti OR malignan*:ab,ti
	#3	#1 AND #2
elastography/stiffness	#4	stiff*:ab,ti OR elastic*:ab,ti OR elastogra*:ab,ti OR fibroscan:ab,ti OR acoustography:ab,ti OR acoustographies:ab,ti OR sonoelastography:ab,ti OR sonoelastographies:ab,ti OR 'acoustic radiation force impulse':ab,ti OR arfi:ab,ti
lymph node/metastasis	#5	lymph:ab,ti OR lymphatic:ab,ti OR metastases:ab,ti OR metastasis:ab,ti
Final search strategy	#6	#3 AND #4 AND #5

Final record: 503 studies.

Fig S1 Quality assessment graphs of the included quantitative elastography studies according to QUADAS-2 guidelines in respect to the following four aspects: patient selection, index test, reference standard and flow and timing. The risk degrees of bias were classified as high (red), unclear (yellow) or low (green). (a) Risk of bias and applicability concerns of each included research. (b) Proportion and numbers of researches with risk of bias and applicability concerns

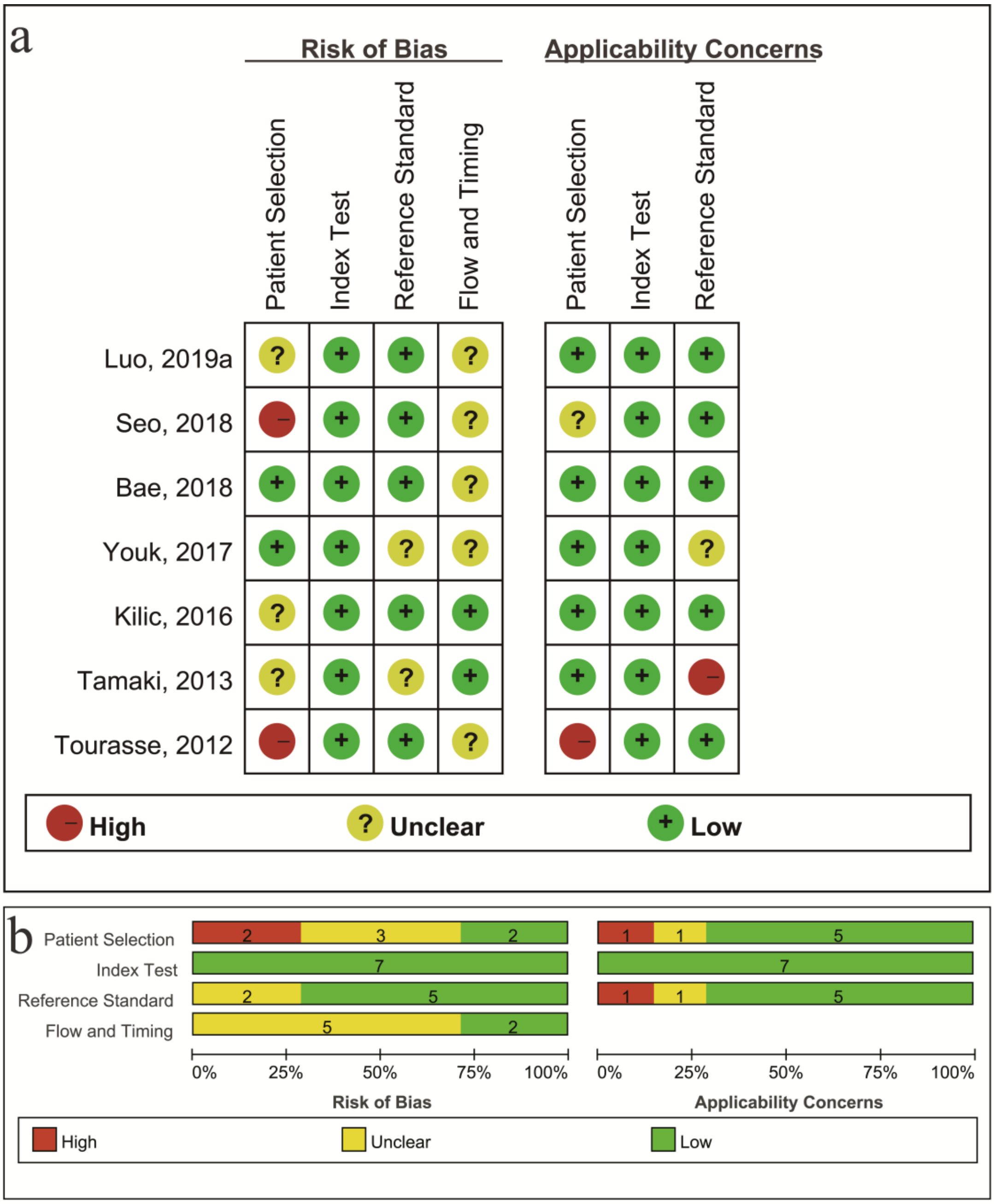

Fig S2 Quality assessment graphs of the included qualitative elastography studies according to QUADAS-2 guidelines in respect to the following four aspects: patient selection, index test, reference standard and flow and timing. The risk degrees of bias were classified as high (red), unclear (yellow) or low (green). (a) Risk of bias and applicability concerns of each included research. (b) Proportion and numbers of researches with risk of bias and applicability concerns

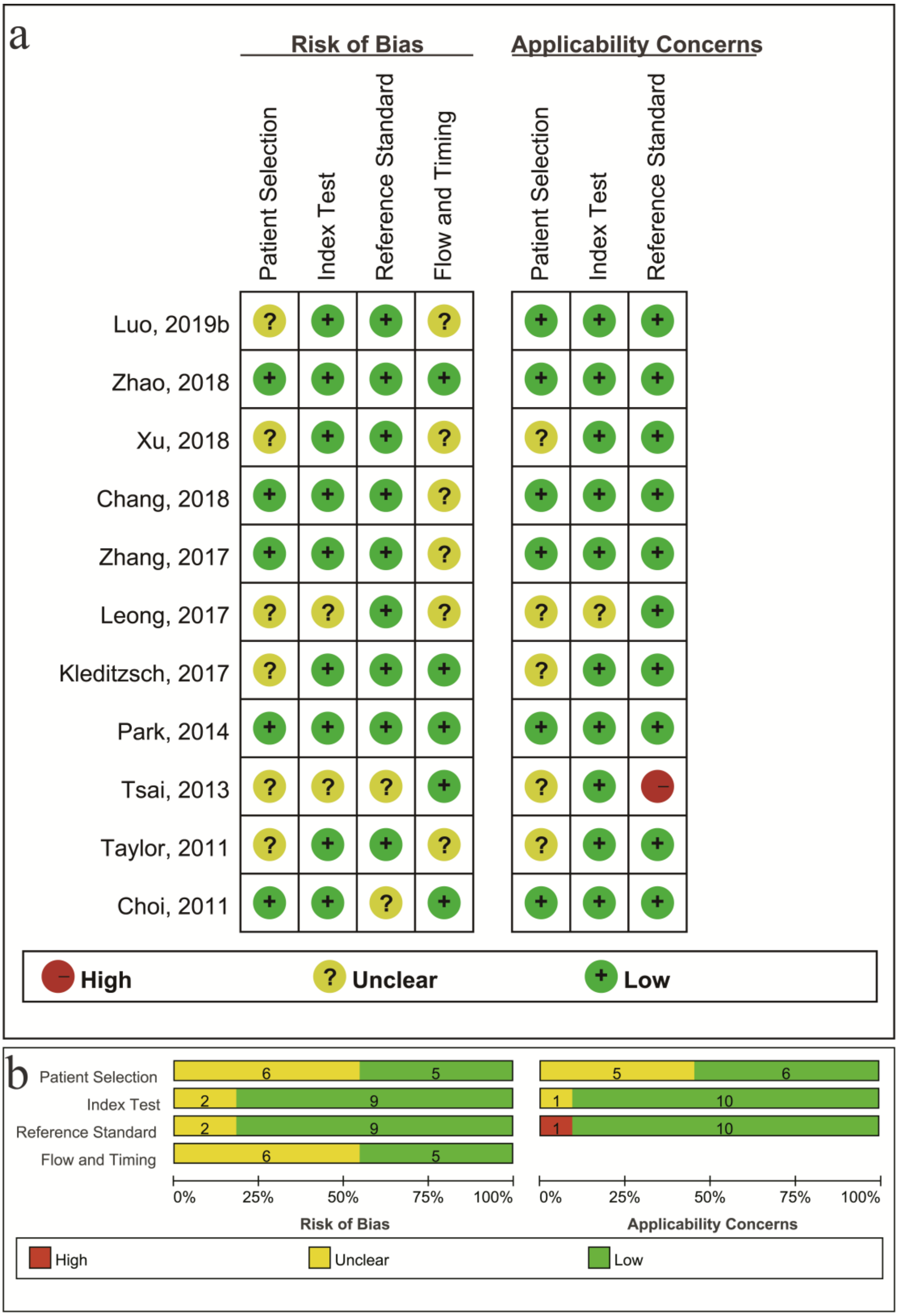

Supplement: Supplementary file 1 [file Data_Sheet_1.PDF]
